# JUVENILE LOCALIZED SCLERODERMA WITH AUTOIMMUNE THYROID DISORDER

**DOI:** 10.4103/0019-5154.70701

**Published:** 2010

**Authors:** N C Hiremath, N T Madan Mohan, C Srinivas, Prabhakar M Sangolli, K Srinivas, V D Vrushali

**Affiliations:** *From the Department of Dermatology, Dr. B. R Ambedkar Medical College and Hospital, Bangalore - 560 045, Karnataka, India. E-mail: pmsangolli@rediffmail.com*

Sir,

When scleroderma of any type afflicts children, it is called Juvenile scleroderma.[1] They constitute about 10% of patients suffering from scleroderma. The age of onset is six to eight years. In the pediatric age group, localized scleroderma is much more common than systemic scleroderma.[2]

An 11-year-old girl, a product of *Non consanguinous* marriage, presented with multiple scaly lesions on trunk and limbs of six years duration. There was no history of trauma, infection or vaccination prior to onset of lesions. Previous treatment was unsatisfactory. All first degree relatives were normal.

**Figure 1:**
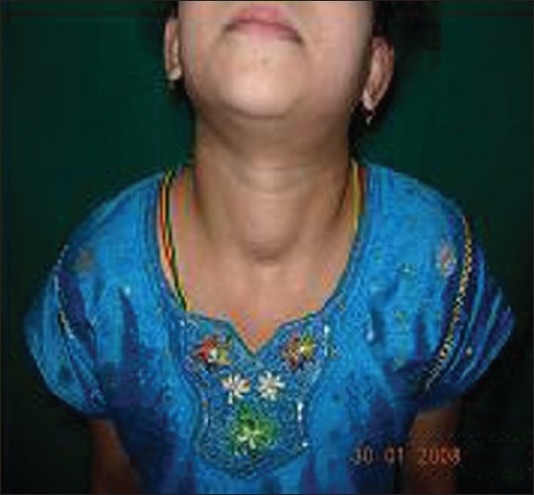
Solitary swelling in neck

**Figure 2:**
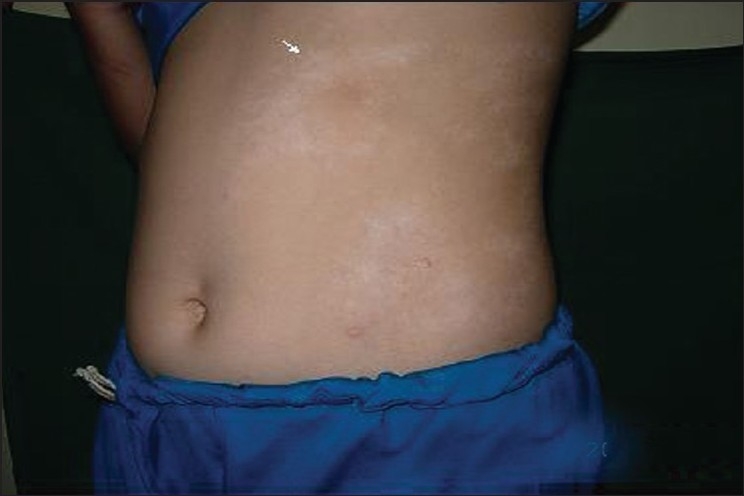
Streaks of depigmentation on abdominal wall along Blaschko’s lines

**Figure 3:**
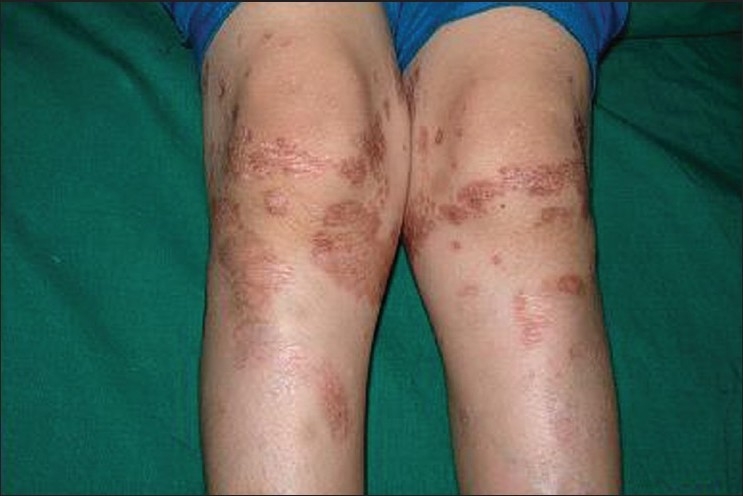
Erythematous scaly plaques on lower limbs

General and systemic examination was unremarkable except for a solitary soft swelling in anterior neck which moved upward on deglutition [Figure 1]. On cutaneous examination, multiple atrophic scaly plaques and guttate papules were seen on trunk and limbs [Figure 2]. There were multiple streaks of depigmentation on anterior abdominal wall along Blaschkos’ lines [Figure 3].

Clinical differential diagnoses of morphea and lichen sclerosis were considered. Routine investigations were normal. Serum T3/T4 levels were normal Serum TSH was markedly elevated-150 IU/ml (normal value is 5-10 IU/ml). Serum anti-thyroglobulin antibody titer was normal. However, antithyroid peroxidase antibody titers were significantly elevated-160 IU/ml (normal value: <40 IU/ml)). Skin biopsy had features consistent with diagnosis of morphea. Fine needle aspiration cytology(FNAC) of neck swelling revealed features of colloid goiter. Testing of RA factor and ANA revealed no abnormality. Final diagnosis of juvenile scleroderma with autoimmune thyroid disease was made. Patient received 25micrograms of L-thyroxine/day. She was treated with topical steroids and weekly oral methotrexate (0.3mg/kg/wk), for morphea, with satisfactory improvement.

Childhood morphea (juvenile scleroderma) constitute 10% of cases of scleroderma. Co-existence of two different morphological patterns is reported in 15% of patients, as in our patient who had guttate as well as plaque type of lesions.[3] Various dermatological conditions are seen in morphea patients. Our patient had clinical features suggestive of nevus depigmentosus. Vitiligo, another auto immune disorder could not be ruled out as biopsy from depigmented lesions could not be performed. Vitiligo has been reported in patients with morphea and Hashimoto’s thyroiditis.[4]

Some studies have indicated an association of autoimmune diseases and an increased frequency of serum auto antibodies in patient with morphea. Harrington and Dunsmore found different autoimmune diseases (pernicious anemia, hypothyroidism, thyrotoxicosis and diabetes mellitus) in eight of their 50 morphea patients[5] The simultaneous occurrence of morphea and autoimmune thyroiditis has been reported by several authors.[6,7] However, all their patients belonged to the adult age group. Thyroid hormones act on nuclear receptors of human fibroblasts and intermediate the regulation of collagen synthesis and degradation. The co-existence of two autoimmune diseases in patients may not have occurred by chance, but could be attributed to autoimmune reactions elicited by the recognition of common antigens. The existence of a separate subset of scleroderma with autoimmune thyroiditis is another possibility which has to be seriously explored.[8] Juvenile scleroderma with autoimmune thyroid disorder has not been reported in Indian literature which prompted us to send this case report.
